# Free-Standing Self-Assemblies of Gallium Nitride Nanoparticles: A Review

**DOI:** 10.3390/mi7090121

**Published:** 2016-08-23

**Authors:** Yucheng Lan, Jianye Li, Winnie Wong-Ng, Rola M. Derbeshi, Jiang Li, Abdellah Lisfi

**Affiliations:** 1 Department of Physics and Engineering Physics, Morgan State University, Baltimore, MD 21251, USA; roder1@morgan.edu (R.M.D.); Abdellah.Lisfi@morgan.edu (A.L.); 2 Department of Materials Science and Engineering, University of Wisconsin-Madison, Madison, WI 53706, USA; jianyeli@hotmail.com; 3 Materials Science Measurement Division, National Institute of Standards and Technology, Gaithersburg, MD 20899, USA; winnie.wong-ng@nist.gov; 4 Department of Civil Engineering, Morgan State University, Baltimore, MD 21251, USA; Jiang.li@morgan.edu

**Keywords:** self-assembly, nanoparticles, Gallium nitride (GaN), renewable energy, review

## Abstract

Gallium nitride (GaN) is an III-V semiconductor with a direct band-gap of 3.4eV. GaN has important potentials in white light-emitting diodes, blue lasers, and field effect transistors because of its super thermal stability and excellent optical properties, playing main roles in future lighting to reduce energy cost and sensors to resist radiations. GaN nanomaterials inherit bulk properties of the compound while possess novel photoelectric properties of nanomaterials. The review focuses on self-assemblies of GaN nanoparticles without templates, growth mechanisms of self-assemblies, and potential applications of the assembled nanostructures on renewable energy.

## 1. Introduction

Gallium nitride (GaN) based semiconductors have attracted great attentions since the 1990s [1,2,3,4]. GaN is a binary III–V direct band-gap semiconductor. Wurtize GaN has a space group of P$6_{3}$mc with lattice constants a=3.1891(1)Å and c=5.1853(3)Å [5]. Its wide band gap of 3.4eV [6,7] affords the nitride special properties for applications in optoelectronic, high-power and high-frequency devices, high-temperature microelectronic devices, and bright lighting sources. For example, (1) hexagonal GaN crystalline films have been fabricated as blue light emitting diodes (LEDs) [3,4,8,9,10] because of its special optical emission [11,12]. The alloyed InGaN- and AlGaN-based LEDs can emit colorful light from red to ultra-violet [9]; (2) GaN films have been employed to make violet (about 405nm) laser diodes (LDs) [3,4], without use of nonlinear optical frequency-doubling; (3) The first GaN-based metal-semiconductor field-effect transistors (MESFET) were experimentally demonstrated in 1993 [13] and commercially available in 2010. High-speed field-effect transistors and high-temperature microelectronic devices were also developed using the material [2] in the 1990s; (4) GaN high-electron-mobility transistors (HEMTs) have been commercialized since 2006, applied at high efficiency and high voltage operation. GaN transistors can operate at much higher temperatures and work at much higher voltages than gallium arsenide transistors. GaN HEMTs are ideal power amplifiers at microwave frequencies; (5) GaN materials have also been utilized as renewable energy materials, such as in thermoelectric devices [14,15,16,17] to harvest waste heat, betavoltaic microbatteries [18,19,20] to collect energy from radioactive sources emitting beta particles, and solar cells [21] to collect solar energy. More applications of GaN materials can be found in some review literatures [1,2,4,22].

GaN is not sensitive to ionizing radiation, making it a suitable material working in radiation environments. Therefore, the GaN-based devices, including LEDs, LDs, MESFETs, HEMTs, and solar cells, have valuable applications in military and out space activities, showing stability in radiation environments.

To date, GaN nanoparticles [23], nanorods [24,25,26,27,28,29,30,31], nanowires [32,33,34,35,36,37,38,39,40,41], and nanotubes [24,42,43,44,45,46,47] have been synthesized using a variety of techniques besides single crystals [48,49,50,51,52]. Compared with GaN crystalline bulks and films, GaN nanomaterials usually show tuning optical properties [53] and more interesting behaviors because of the quantum confinement, having wider applications [54] than bulks and films. Individual crystalline nanorods and nanowires have been utilized as nano-LEDs [55], LDs [56,57,58], and field effect transistors [59,60]. Assemblies of nanorods and nanowires have also been fabricated into nanowire LEDs [61,62,63], nanorod LEDs [64], and nano generators [65,66].

GaN nanoparticles have the highest surface / volume ratio among all GaN nanomaterials. Here we focus on free-standing self-assemblies of GaN nanoparticles. Syntheses and physical properties of GaN nanoparticles will be introduced, followed free-standing self-assemblies of GaN nanoparticles. The unique physical properties and potential applications of the free-standing assemblies are discussed at the end.

## 2. Syntheses of GaN Nanoparticles

Hexagonal GaN nanoparticles have been synthesized by various techniques. Among these techniques, chemical vapor deposition, nitridation, solvothermal technique, and ball-milling are popular methods to produce GaN nanoparticles. Table 1 compares these four kinds of methods.

### 2.1. Chemical Vapor Deposition

GaN nanoparticles can be easily prepared from a reaction of gallium and ammonia by chemical vapor deposition (CVD) methods at 900–1150 ${}^{\circ }C$ [5,49,67,68,75], and from organic gallium compounds by detonations or pyrolysis [76,77,78].

**Figure 1:**
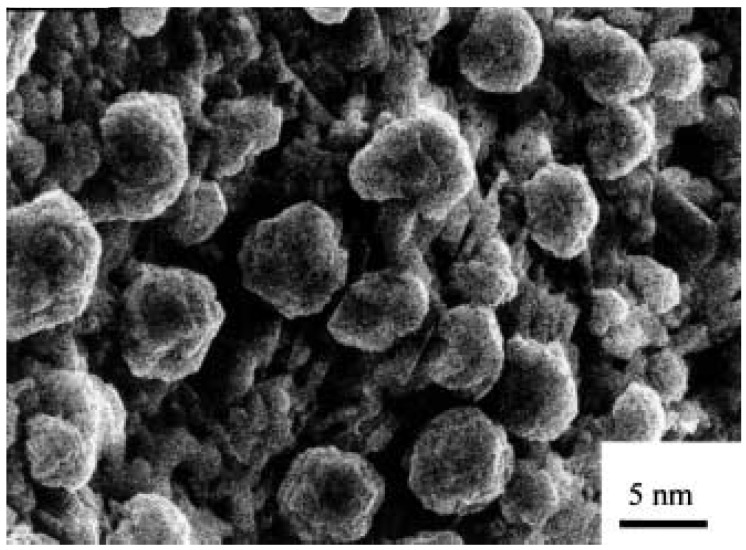
GaN nanoparticles synthesized by gas reactions on rough LaAlO${}_{3}$ substrates. Reprinted with permission from [67]. Copyright 2000 Springer.

Figure 1 shows spherical GaN particles synthesized from gallium by a CVD method. Gallium gas reacted directly with ammonia to form GaN nanoparticles. The diameters of the nanopaticles were about 5–8 nm, smaller than the exciton Bohr radius of about 10nm.

GaN nanoparticles were recently successfully synthesized via an microwave plasma-enhanced CVD method [79]. The resulting GaN nanoparticles had an average size of around 8.5nm with a very narrow size distribution.

GaN nanocrystals were also prepared from polymericgallium imide, (Ga(NH)${}_{3/2}$)${}_{n}$, and trioctylamine at $360{\;}^{\circ }C$ [80]. The produced GaN nanocrystals were spherical with a mean diameter of 30±12Å.

The band gap of the GaN nanocrystals usually shifts slightly to higher energy because of the quantum confinement. The detailed optical properties of GaN nanoparticles will be discussed in Section 3.

The CVD synthesized GaN nanoparticles usually crystallized well because of higher reaction temperature. However, there are nitrogen- or gallium-vacancies in CVD-grown GaN nanoparticles, affecting optical properties. The details will be discussed in Section 3.

It is challenging to produce massive GaN nanoparticles using the CVD methods because of high cost of starting materials and low product yields.

### 2.2. Nitridation

Single-phase and high-pure GaN nanoparticles have been produced through nitridation of Ga-based materials. The Ga-based precursors are usually cheaper than gallium and GaN.

It was reported that GaN nanoparticles were achieved via a reaction of gallium oxide with ammonia [68,81,82] over $1100{\;}^{\circ }C$ in flowing ammonia. Pure GaN nanoparticles were synthesized through a nitridation reaction as expressed [68]:

(1) $${\mathrm{Ga}}_{2}O_{3}\left(s\right)+2{\mathrm{NH}}_{3}\left(g\right)\to 2\mathrm{GaN}\left(s\right)+3H_{2}O\left(g\right)$$

The crystalline size of the produced GaN nanoparticles strongly depended on particle size of the Ga${}_{2}$O${}_{3}$ starting materials. Therefore, it is critical to employ nanoscale precursors to produce GaN nanoparticles. Additionally, the nitridation temperature varies with grain size of precursors. It was reported that the nitridation temperate was lowered to $800{\;}^{\circ }C$ when the grain size of gallium oxide nanoparticles was less than 20nm [82]. Ga${}_{2}$O${}_{3}$ is much cheaper than Ga and the total cost of GaN nanoparticles would be reduced.

Besides Ga${}_{2}$O${}_{3}$ powders, other gallium-based materials, such as gallium phosphide GaP microcrystalline particles [83], gallium antimonide GaSb powders [69], gallium arsenide GaAs powders [84], and gallium nitrate Ga(NO${}_{3}$)${}_{3}$ powders [70], were utilized to produce pure GaN nanoparticles under ammonia NH${}_{3}$ at high temperatures in one step nitridation procedures. The chemical reaction of the nitridation procedures was similar to Equation (1).

A soluble salt nitration technique was recently developed [85] to synthesize GaN nanoparticles at low temperatures. Ga metal was mixed with N${}_{2}$SO${}_{4}$ powders and then heated at $700{\;}^{\circ }C$ under NH${}_{3}$/N${}_{2}$ atmosphere. The weight ratio of Ga and Na${}_{2}$SO${}_{4}$ was 1:10, which was adequate to obtain high dispersion of Ga (melting point of $30{\;}^{\circ }C$) droplets onto Na${}_{2}$SO${}_{4}$ (melting point of $884{\;}^{\circ }C$) powders. GaN nanoparticles with mean diameter of 7.6nm were produced in a large scale with a high yield (95.8%) through a direct nitridation reaction. Na${}_{3}$PO${}_{4}$ was also utilized as a dispersant [86] in the soluble salt-assisted technique. Large scaled high-crystalline GaN nanoparticles were produced through a direct nitridation of Ga-Na${}_{3}$PO${}_{4}$ mixture at 750–950 ${}^{\circ }C$. The mean diameter of the produced nanoparticles was 8–18 nm, depending on nitridation temperature.

Gallium oxide hydroxide was also employed as a precursor to produce GaN nanoparticles [71]. GaO(OH) nanoparticles were synthesized from a chemical co-precipitation method, and heated at high temperatures under ammonia. The average particle size of produced GaN powders was 12–15 nm when the nitridation temperature was 900–1000 ${}^{\circ }C$.

Nitridation is an economically effective method to synthesize massive GaN nanoparticles.

### 2.3. Solvothermal Techniques

Solvothermal methods can synthesize various materials under supercritical conditions at high temperatures and high pressures [87]. Based on the used solvent type, the method is termed as hydrothermal (water as solvent), ammonothermal (ammonia as solvent), or benzene-thermal (benzene as solvent) method.

Ammonothermal technique is an effective method to produce massive GaN nanoparticles from gallium at low temperatures [23,88]. Nanocrystalline GaN powders were synthesized under base [23,48] or acidic conditions [72,89] using various mineralizators.

It was reported that gallium nitride nanoparticles were synthesized from gallium metal in stainless steel autoclaves at 350–500 ${}^{\circ }C$ in the presence of lithium mineralizator (pressure about 2000atm at reaction temperatures) [23], potassium mineralizator [48], sodium mineralizator [48], and LiNH${}_{2}$ mineralizator [48]. The ammonia solutions were base because of the mineralizators. The average grain size of GaN powders was about 32nm or less. When lithium was used as a mineralizator, alkali metal amide LiNH${}_{2}$ was produced in the solvent:

(2) $$2{\mathrm{NH}}_{3}+2\mathrm{Li}\to 2{\mathrm{NH}}_{2}^{-}+H_{2}+2{\mathrm{Li}}^{+}$$

to form an ammono-basic solution. It was believed that intermediate compounds such as LiGa(NH${}_{2}$)${}_{4}$ were formed during the ammonothermal procedure and followed a reaction [23]:

(3) $$\mathrm{LiGa}\left({\mathrm{NH}}_{2}\right){}_{4}\to \mathrm{GaN}+{\mathrm{LiNH}}_{2}+2{\mathrm{NH}}_{3}$$

The chemical reaction was similar when potassium or sodium was employed as mineralizator. It was believed that alkali metal amide, Na[Ga(NH${}_{2}$)${}_{4}$] [90] or K[Ga(NH${}_{2}$)${}_{4}$] [91], was produced under the supercritial base-conditions. The intermediate compounds converted to GaN during ammonothermal procedures:

(4) $$A\left[\mathrm{Ga}\left({\mathrm{NH}}_{2}\right){}_{4}\right]↔\mathrm{GaN}+{\mathrm{ANH}}_{2}+2{\mathrm{NH}}_{3}$$

where A is Na or K.

Nanocrystalline GaN powders were also synthesized under acidic conditions [72,89]. Ammonium halides NH${}_{4}$X (X = F, Cl, Br, I) were typical ammonoacidic mineralizers to synthesize GaN in ammonothermal procedures. The ammonium halides increased concentration of ammonium ions NH${}_{4}^{+}$. Intermediate hexammoniates, such as [Ga(NH${}_{3}$)${}_{6}$]Br${}_{3}$·NH${}_{3}$ and [Ga(NH${}_{3}$)${}_{6}$]I${}_{3}$·NH${}_{3}$ [92], would form in ammonia and the produce Ga-containing ions mobiled in solutions. The Ga-containing ions decomposed to GaN nanoparticles at high temperatures [93].

GaN nanocrystaline particles can also be produced by a benzene-thermal synthetic route [73,94]. A benzene-thermal reaction of Li${}_{3}$N and GaCl${}_{3}$ was carried out [73] in which benzene was used as the solvent under high pressures at $280{\;}^{\circ }C$. The size of the prepared GaN nanoparticles was 30nm. The reaction temperature was much lower than that of traditional methods, and the yield of GaN reached 80%. The X-ray powder diffraction pattern indicated that the produced nanomaterials was mainly hexagonal-phase GaN with a small fraction of rocksalt-phase GaN. GaN nanoparticles were also synthesized at room temperature [95] when Li${}_{3}$N and GaCl${}_{3}$ was mixed with a ratio of 1:1 and kept in benzene and ether for hours. Ether greatly accelerated the reaction.

GaN spherical nanoparticles were also synthesized in superheated toluene [96]. Gallium chloride reacted with sodium azide in toluene to produce insoluble azide precursors. The produced precursors solvothermally decomposed to GaN at temperatures below $260{\;}^{\circ }C$. The resulting GaN nanomaterials were poorly crystalline but thermally stable.

Recently GaN nanoparticles were prepared in a tubular microreactor at supercritical conditions [97] using cyclohexane or hexane-ammonia solutions as solvents (temperature: ∼450 ${}^{\circ }C$ and reaction time: <25 s). The as-prepared nanoparticles exhibited a strong luminescence in the ultraviolet.

### 2.4. Ball-Milling Techniques

Ball-milling is an effective industrial technique to produce massive GaN nanoparticles. Dependent on the type of ball-mills [22], various nanoparticles can be produced by mechanical grinding or mechanical alloying.

GaN nanoparticles were also produced directly from GaN materials [28,98] by high-energy ball-milling through mechanical grinding. The particle size was reduced significantly after ball-milling, confirmed by X-ray diffraction and dynamic light scattering.

Gallium nitride powders were synthesized by mechanical alloying of gallium metal in a dry ammonia atmosphere at elevated temperatures (about $100{\;}^{\circ }C$) [74]. Transmission electron microscopy (TEM) indicated that the diameter of obtained GaN nanocrystals ranged between 10 and 30 nm. Larger crystals with a size around 70 nm were also observed.

GaN nanoparticles were also synthesized by mechanochemical reaction between Ga${}_{2}$O${}_{3}$ and Li${}_{3}$N [99] through ball-milling under ammonia gas environment. The molar ratio of Ga${}_{2}$O${}_{3}$ and Li${}_{3}$N was 1:2. GaN nanopowders were prepared within 2 h with a mill speed of 300 rpm at room temperature.

### 2.5. Other Techniques

Many other techniques have been developed to produce GaN nanoparticles. For examples, GaN nanoparticles were produced by pulsed laser ablation [100]. High quality GaN nanocrystalline were prepared by sol-gel method [101] with size of 30–100 nm. GaN nanoparticles were also produced from organometallic gallium azides at low temperature, such as $216{\;}^{\circ }C$ [102], in solutions. The crystalline quality of the produced materials was very poor while crystallized after annealing at $220{\;}^{\circ }C$.

Among these techniques, CVD method can produce high quality GaN nanoparticles while is cost. Ammonothermal technique, nitridation method, and ball-milling method can produce massive GaN nanoparticles economically.

## 3. Physical Properties and Applications of GaN Nanoparticles

### 3.1. Physical Properties of Crystalline Nanoparticles

According to the classical thermodynamics, the energy band gap of GaN nanomaterials depends on crystalline size:

(5) $$E_{nano}=\left(1+\frac{\alpha }{D}\right)E_{bulk}$$

where the shape parameter α=0.462 for spherical particles. The confinement-dependent exception binding energy of GaN nanoparticles was detected experimentally [103] from photoluminescence. The energy shifted to higher energy with decreasing particle size, in agreement with the theoretical prediction.

The size-dependent band-gap was confirmed experimentally from photoluminescence of GaN nanoparticles. The photoluminescence spectrum of GaN nanoparticles usually blue-shifted [67,104,105] compared with that of GaN bulks (365nm), which was ascribed to the quantum confinement effect. It was reported the band gap of the GaN nanocrystals shifted slightly to higher energy [80] once the diameter was about 30nm. Its band edge emission with several emission peaks appeared in the range between 3.2 and 3.8eV at low temperature while there were two excited-state transitions at higher energies [80].

The melting temperature of GaN nanoparticles depends on the crystalline size too [106,107]. Numerical thermodynamical approach predicated the melting point of spherical GaN nanoparticles was 100–200 K lower than that of bulks.

The size of GaN nanoparticles has great influence on dielectric behaviors of GaN nanoparticles [108]. Due to the existence of interfaces with a large volume fraction, hexagonal GaN nanoparticles have much higher dielectric constant than that of hexagonal GaN coarse-grain powders.

The radiative decay time of excitons also depends on the GaN nanoparticle size [109]. Theoretical simulations indicated that GaN quantum dots had a long radiative decay time and was undesirable for optoelectronic applications as light-emitting diodes [110]. Therefore, GaN nanoparticles should be good candidates for the development of light emitting diodes, laser diodes, and novel optical devices in the short-wavelength region with a wide-wavelength tuning range [109].

### 3.2. Defects of GaN Nanoparticles

There are four kinds of defects in GaN, zero-dimensional point defects, one-dimensional dislocations, two-dimensional planar defects, and three-dimensional volume defects. These defects affect optical, electrical, mechanical and thermal properties of gallium nitride, such as introducing strains in GaN materials, changing lattice constants and band-gap energies, forming donor or acceptor levels in band gaps.

Up till now, few reports have discussed these defects of GaN nanoparticles. Compared with crystalline bulks and films, GaN nanoparticles have a super high surface/volume ratio. The ratio is high up to 0.6 for a 10nm diameter GaN crystalline spherical particle. The high surface/volume ratio would greatly reduce dislocation densities. When GaN nanoparticles are produced at high temperatures (such as 800–1000 °C in CVD and nitridation procedures), there are few three-dimensional defects produced during syntheses. Based on the facts of syntheses conditions and high surface/volume ratio, zero-dimensional defects and two-dimensional grain boundaries would dominate the defect effects of high-temperature grown GaN nanoparticles.

Solvothermal grown GaN nanoparticles should have less defects, similar to ammonothermal grown GaN bulks [48,52]. CVD and nitridation grown GaN particles should produce Ga-vacancy defects or nitrogen-vacancy defects depending on nitrogen-rich or nitrogen-poor environments during high temperature procedures. Ball-milled nanoparticles should have more dislocations and planar defects because of mechanical collisions.

### 3.3. Defect Effects on Physical Properties

Physical properties of nanoparticles are affected by various defects. Three kinds of defects (vacancies, surface defects, and grain boundaries) are mainly discussed below.

**Figure 2:**
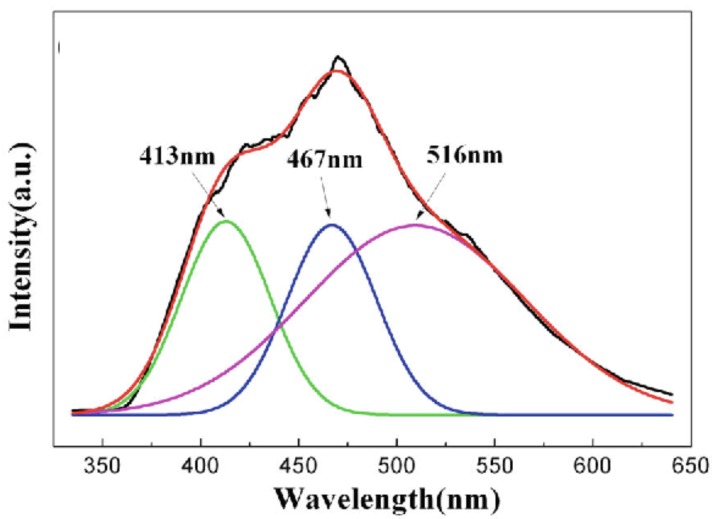
Room-temperature photoluminescence spectrum of GaN nanoparticles with the Ga/N ratio of 0.90. Reprinted with permission from [112]. Copyright 2014 Springer.

Ga vacancies always exist in GaN nanoparticles. Figure 2 shows a PL spectrum of GaN nanoparticles upon excitation at 325nm recorded at room temperature. The diameter of the nanoparticles was 100–200 nm and the Ga/N ratio was 0.90. The spectrum exhibited three distinct features: a blue emission at 413nm coming from the free excitonic transition between valence and conduction bands of stoichiometric GaN, a broad green luminescence(GL) centered at 467nm, and a yellow luminescence (YL) band emission centered at 516nm. The YL peak around 516nm was saturated easily with excitation intensity, indicating the YL band should be attributed to Ga vacancy at surfaces [111]. The GL band at 467nm showed almost no saturation, implying the band was related to isolated Ga vacancy (V${}_{Ga}$).

**Figure 3:**
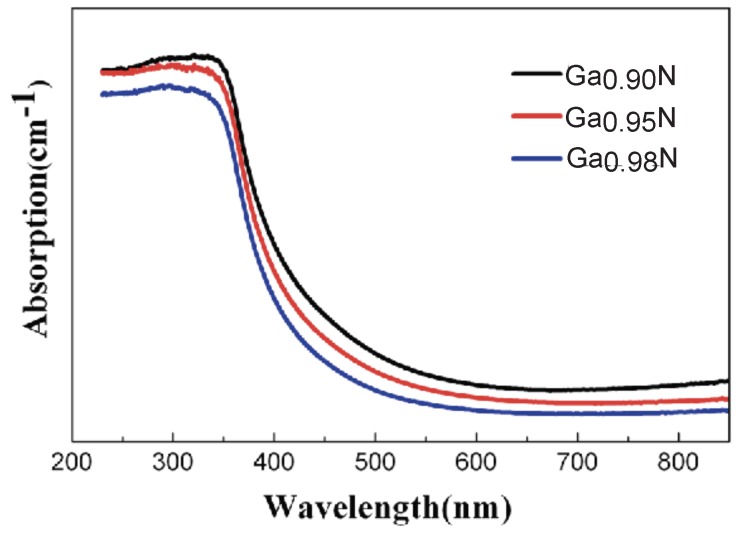
UV-vis spectra of three GaN samples with different concentrations of Ga vacancies. Reprinted with permission from [112]. Copyright 2014 Springer.

More detailed experiments showed that the optical absorption edge moved to longer wavelengths (red-shifted) as the concentration of Ga vacancies increased [112]. Figure 3 shows the effect of Ga vacancy on UV-vis spectrum of GaN nanoparticles. The band-gap of GaN nanoparticles increased with decreasing concentration of Ga vacancy. The band-gap of Ga${}_{0.90}$N nanparticles with the concentration of Ga vacancy of 2.8% was 3.15eV, 3.17eV for Ga${}_{0.95}$N nanoparticles with the concentration of Ga vacancy of 1.8%, and 3.20eV for Ga${}_{0.98}$N nanoparticles with the concentration of Ga vacancy of 1.6% [112].

Nitrogen vacancies were observed in some GaN nanoparticles synthesized under nitrogen-absence environments [113]. The dielectric properties of these N-deficient GaN nanoparticles exhibited significant enhancement over that of GaN nanomaterials at low frequency range.

Doping can induce various defects in GaN nanoparticles and affect optical, magnetic properties. Here we do not discuss it.

Surface defects also affect photoluminescence [32,114]. Similar to Ga vacancies or nitrogen vacancies just discussed, the surface defects changed band gaps and shifted optical absorption edges of GaN nanoparticles.

Surface defects also affect magnetic properties of GaN nanomaterials. GaN bulks are diamagnetic. However, un-doped GaN nanoparticles showed ferromagnetic at room temperature [115] when the average diameter of the nanoparticles was in the range 10–25 nm. Furthermore, the saturation magnetic moment decreased with increasing nanoparticles size, suggesting that ferromagnetism was due to the surface defects of the nanoparticles. Recently experiments indicated the ferromagnetism should be induced by Ga vacancies [112].

Among two-dimensional defects of GaN nanoparticles, grain boundaries play a more important role than stacking faults, twins and inversion domain boundaries. The effects of grain boundaries can be described as the size effect and surface defects, as discussed above. Their effects on optical properties can be described by Equation (5).

Dislocations in GaN materials have played an important role of optical properties, especially for GaN films and GaN bulks. Here the effect is not discussed because the one-dimensional defects do not dominate physical properties of GaN nanoparticles.

Three-dimensional defects, such as voids, cracks and nanopipes are usually observed in bulks and films. However, this kind of defects is rarely observed in nanoparticles and not discussed here.

More detailed information of luminescence properties of defects in GaN can be found in previous review literatures [116].

### 3.4. Applications of Nanoparticles

GaN nanoparticles have been deposited on substrates as solar cells [117]. The conversion efficiency is 3.10% under air mass 1.5 global illumination and room temperature conditions. A further increase of 15% was achieved in short circuit current density, improving the conversion efficiency to 3.87%, in an optimized structure.

GaN nanoparticles with size of 15–20 nm were added to poly(3-hexylthiophene) (P3HT) as hybrid solar cells [118]. With the addition of GaN nanoparticles to P3HT from 5 to 15mg/mL, the performance of the hybrid solar cells was greatly enhanced. The short circuit current density was tripled to $3.5\;mA/cm^{2}$ and filling factor to 44% from 20%.

GaN nanoparticles were employed as photocatalysts for overall water splitting [119]. The photocatalytic activity of GaN for the reaction was found to be strongly dependent on the crystallinity of GaN nanoparitcles.

Compared to bulk materials, GaN nanoparticles have larger surface area, size-dependent properties, increased absorption coefficient, increased band-gap energy, and reduced carrier-scattering rate, offering potential advantages than bulks and films.

## 4. Free-standing Self-assembly of GaN Nanoparticles

GaN nanoparticles were self-assembled into various 2D macroscale structures on substrates, working as LEDs and LDs. 3D free-standing structures, such as porous nanotube arrays [120] and crystalline nanotube arrays [43,121] were also fabricated by templates. Here we focus on free-standing GaN porous materials grown without any templates.

### 4.1. GaN Nanospheres

**Figure 4:**
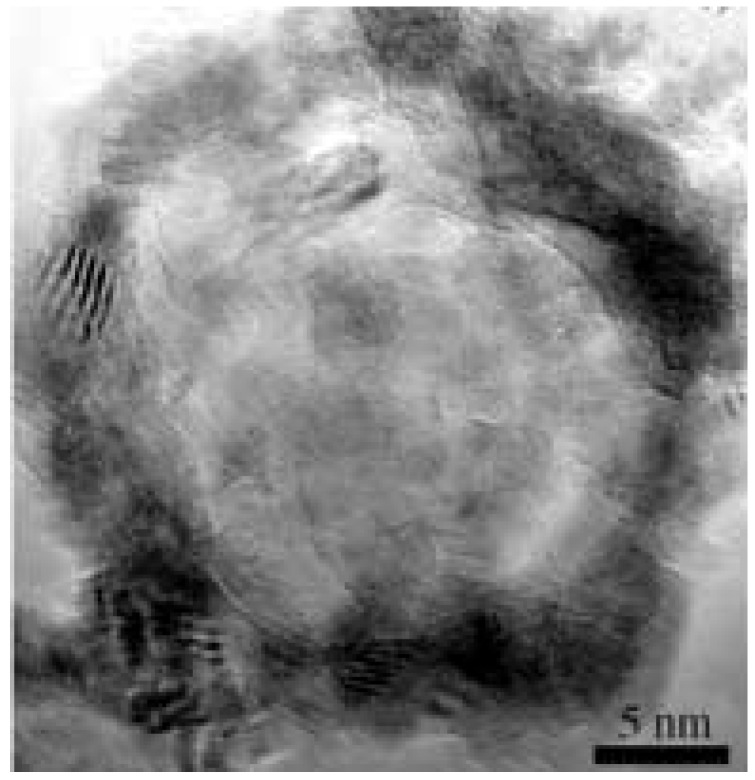
HRTEM image of a GaN nanosphere. Reprinted with permission from [122]. Copyright 2005 WILEY-VCH Verlag GmbH & Co.

Figure 4 shows a GaN nanosphere [122] with a diameter of 20–25 nm. The nanosphere was self-assembled from GaN nanoparticles with a diameter of several nanometers. It was believed that there were lots of gallium nanodroplets around gallium sources. When ammonia NH${}_{3}$ gas was introduced into reaction chambers, NH${}_{3}$ molecules were easily adsorbed onto surfaces of the nanosized gallium droplets. Then GaN nanoparticles nucleated at the surfaces of the metallic gallium nanodroplets. With increasing reaction time, more and more NH${}_{3}$ passed though the previously formed GaN-Ga interfaces to react with Ga to form GaN shells. Hollow spheres were formed when the gallium nanodroplets were consumed. The produced hollow spheres were composed of polycrystalline nanoparticles.

**Figure 5:**
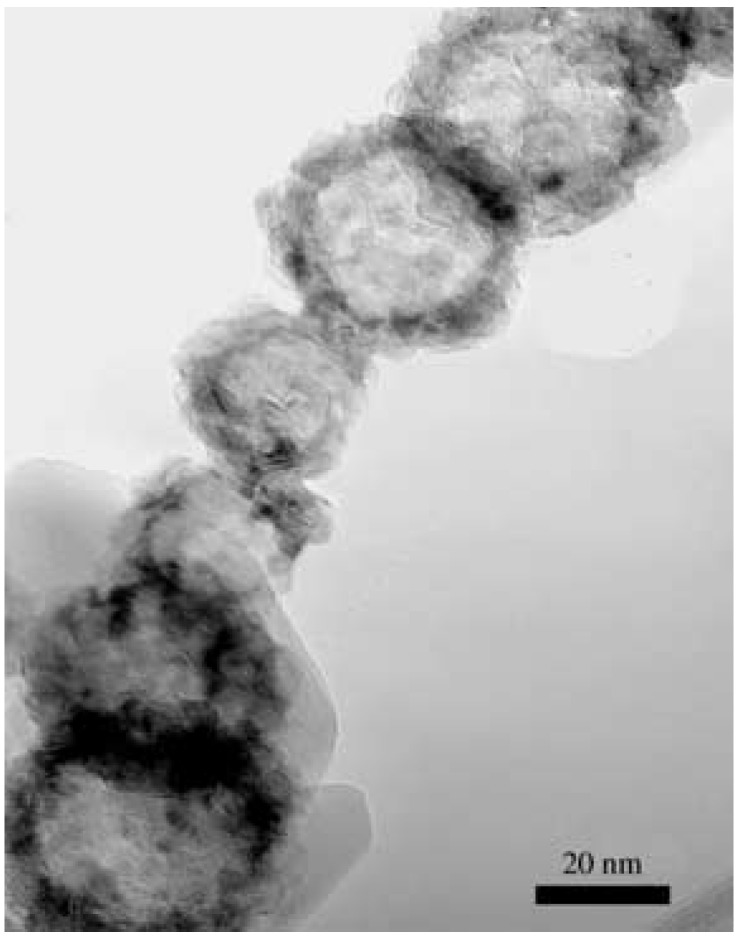
TEM image a GaN sphere column. Reprinted with permission from [122]. Copyright 2005 WILEY-VCH Verlag GmbH & Co.

Sometimes the hollow GaN spheres further aggregated into columns, as shown in Figure 5.

### 4.2. GaN Nanotubes

**Figure 6:**
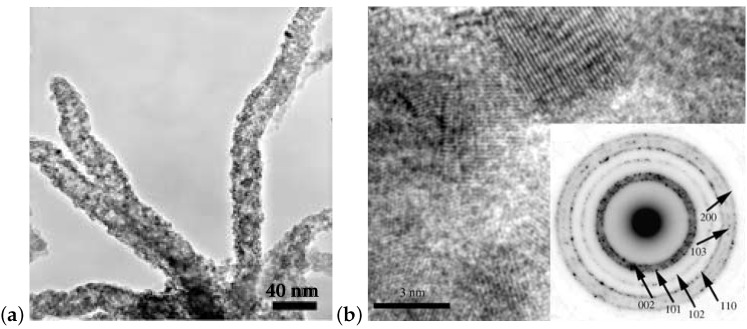
(**a**) Typical TEM image of GaN nanotubes synthesized above $1100{\;}^{\circ }C$ by a gas interface reaction route; (**b**) Cross-sectional HRTEM image of a nanotube. The inset is an SAED pattern of the nanotube. Reprinted with permission from [122]. Copyright 2005 Wiley-VCH Verlag GmbH & Co.

GaN porous nanotubes were self-assembled from GaN nanoparticles [122]. Figure 6a shows several GaN nanotubes. The darker edges indicated that the GaN materials were hollow tubes, not solid rods. The wall thicknesses were 3.5–5.0 nm. The GaN nanotubes had a length of several hundreds of nanometers. Selected area electron diffraction (SAED) patterns (inset of Figure 6b) indicated that the nanotubes were polycrystalline. Figure 6b shows detailed nanostructures of the nanotubes. The nanotube walls were composed of single-layer-ed GaN nanocrystalline particles with a diameter of 3.0–3.5 nm.

It was believed that the hollow GaN nanotubes were formed by the coalescence of the nanosized hollow GaN columns shown in Figure 5.

### 4.3. GaN Circular Microtubes

**Figure 7:**
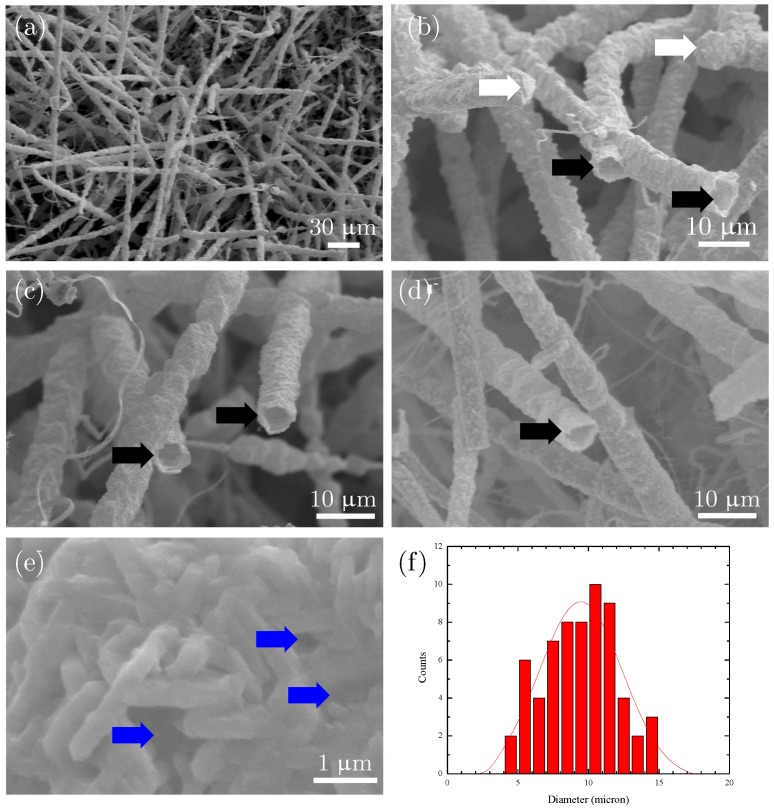
SEM images of (**a**–**d**) GaN microtubes grown on quartz substrates and (**e**) surface of a microtube. White arrows mark the closed ends, black arrows the open ends, and blue arrows the nanopores of the microtubes; (**f**) Histogram of microtube diameter. Reprinted with permission from [123]. Copyright 2015 Elsevier.

Figure 7 shows wurtzite GaN microtubes grown by a CVD method without templates [123]. Gallium was placed on quartz substrates in a hot-walled CVD system. Then Ga reacted with ammonia at 820–840 ${}^{\circ }C$ and yellow GaN nanostructures were produced on the quartz substrates. Scanning electron microscopy (SEM) indicated that the yellow deposits were microtubes (Figure 7a). The diameter of the hollow microtubes was about 8μm and lengths up to 100μm. The ends of most of these microtubes were open, but some were closed (Figure 7b–d).

The thicknesses of the microtube walls were roughly measured from the open ends of the microtubes, about 100nm. Detailed SEM examination (Figure 7e) indicated the walls were consisted of single layer of randomly oriented nanostructures. The diameters of the nanostructures were about 100nm and their lengths were about 1μm.

SEM images (Figure 7e) also indicated that the thin walls of the microtubes were porous because of the random packing of the nanostructures. Some nanopores of these walls are notated by blue arrows in Figure 7e. Such porous microtubes should have large surface areas.

X-ray powder diffraction indicated that the microtubes were hexagonal GaN nanomaterials [123]. The phase was also confirmed by SAED on TEM.

### 4.4. GaN Squared Microtubes

**Figure 8:**
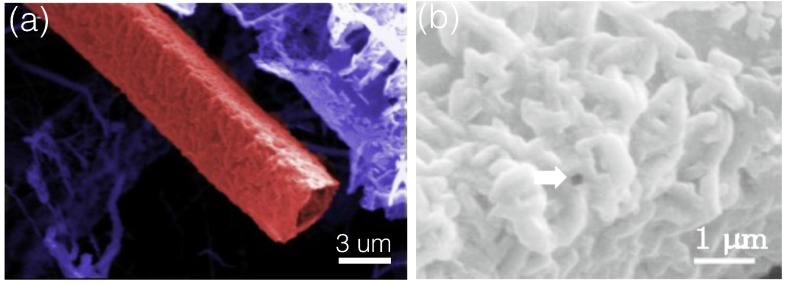
SEM images of (**a**) a squared GaN microtube and (**b**) its surface. Reprinted with permission from [124]. Copyright 2013 Springer.

Squared GaN microtube were also synthesized by the CVD method [124], as shown in Figure 8a. The microtubes were several microns long. The thicknesses of the microtube walls were about 100nm. More detailed SEM images (Figure 8b) indicated that the walls consisted of single layer of random nanoparticles.

**Figure 9:**
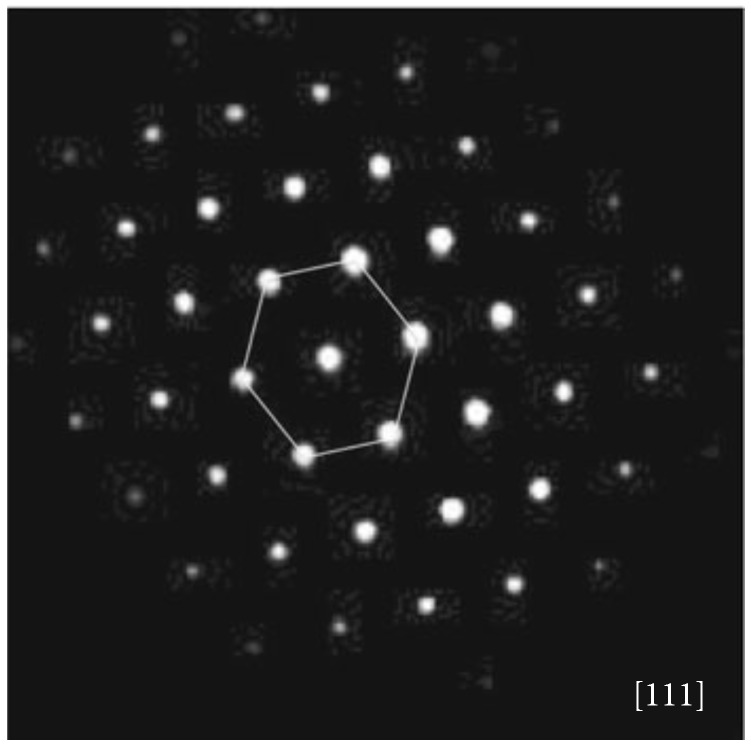
SAED pattern of a nanoparticle consisting of squared GaN microtubes. Reprinted with permission from [124]. Copyright 2013 Springer.

Figure 9 shows a selected area electron diffraction (SAED) pattern taken from a nanoparticle of the squared microtubes. The nanoparticle was single crystalline. The SAED pattern had a three-fold symmetry and was indexed as the cubic GaN phase with the space group of F$\bar{4}$3m along a zone axis of [111] (the lattice parameter a=4.503Å). Therefore, the squared microtubes should be consisted zinc-blende GaN nanoparticles.

The formation of the porous microtubules can be explained by the Liesegang phenominon and diffusion limited growth. Large amounts of gallium liquid droplets condense on quartz substrates from gaseous gallium. Gallium nanodroplets evaporate at high temperatures to produce gallium vapor. Once ammonia passes the gallium nanodroplets, gallium vapor would react with ammonia to produce GaN nanoparticles (nanorods or irregular nanostructures). The number of the GaN nanoparticles per unit volume is so dense around the gallium droplets that the GaN nanoparticles can be considered as a supersaturated aerosol around the gallium droplets. These supersaturated GaN nanoparticles would spontaneously aggregate into Liesegang rings because of thermodynamatic stability [125,126]. GaN nanoparticle Liesegang rings were observed [124] and support the hypothesis.

Then porous GaN microtubules grow on the Liesegang rings. Based on the diffusion-limited aggregation mechanism, the protruding parts of the GaN microrings would easily attract the GaN nanoparticles and grow quickly to build a new nanoparticle-wall from the Liesegang ring edges, leaving the hollow interior.

With the consuming of gallium, supersaturated GaN nanoparticles would be continuously synthesized from the reaction of ammonia and gallium vapor as long as the gallium vapor pressure are high enough, resulting in the continuous deposition of nanoparticles on the GaN microrings to form GaN microtubules. During the whole growth stage, the gallium droplets would evaporate to generate the gallium vapor to keep the microtubules open or the microtubes would be closed if the gallium nano droplets were consumed before the experiments ended.

### 4.5. GaN Nanocomposite Bulks

**Figure 10:**
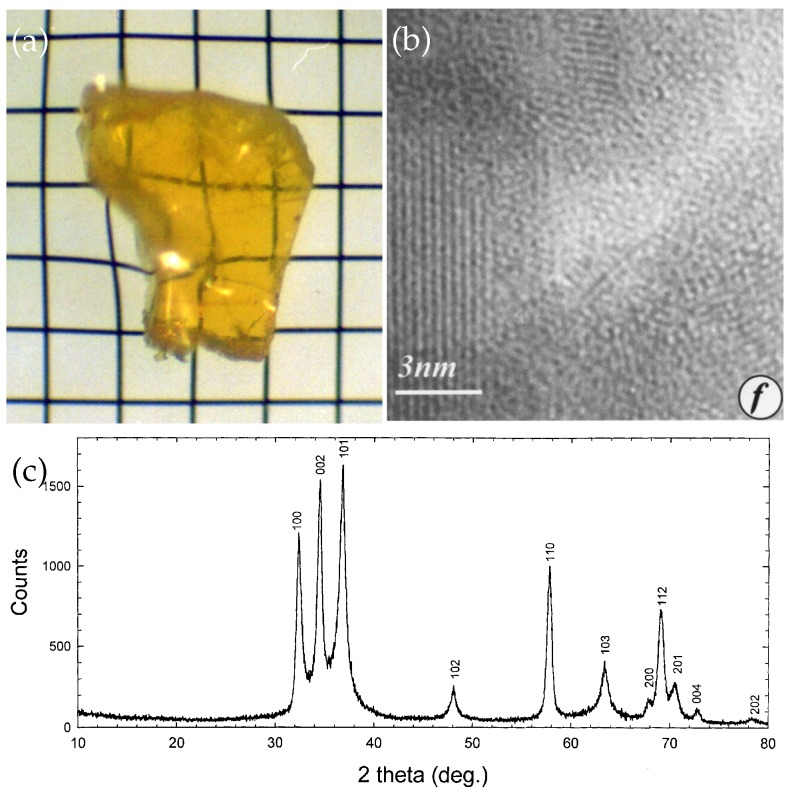
(**a**) Optical photograph of a fragment of GaN nanocomposites. The grid spacings are 0.2×0.2cm${}^{2}$; (**b**) HRTEM image of three adjacent nanograins; (**c**) An XRD pattern of GaN nanocomposites. Reprinted with permission from [72]. Copyright 2000 Elsevier.

GaN nanocomposite bulks were in-situ fabricated under ammonothermal conditions. The GaN nanocomposite was directly produced through reactions of Ga metal and ammonia with NH${}_{4}$Cl catalysts in autoclaves [72]. Figure 10a shows a typical photograph of a fragment of the nanocomposites. The size of the fragment was about 0.7×0.6cm${}^{2}$ with an thickness of about 0.2cm. The fragment was transparent to visible light. SEM examinations revealed the existence of terrace steps which resemble characteristics of brittle materials. The assemblies were bulk materials like polycrystalline ceramic materials, not simple mechanical aggregation of grains.

The GaN nanocomposite bulks were consisted of nanograins with several nanometers. Figure 10b shows an HRTEM image of three adjacent GaN nanograins with [001] orientation. Each adjacent nanograin was chemically bounded with others. Among the three adjacent nanograins, a nanopore was generated. The size of these pores was around 5nm, roughly half of the average diameter of grains. Since the size of pores was well below the wavelengths of visible light, the GaN nanocomposites was transparent to visible light as shown in Figure 10a.

The exist of nanopores in the nanocomposites was indirectly confirmed by mass density measurements. The measured mass density for the GaN nanocomposites was 72% of that for the GaN single crystals. This value approached the theoretical limit of close-packing of equal spheres.

Figure 10c shows the XRD pattern of the GaN nanocomposites. The XRD pattern indicated that the assembly was hexagonal GaN. All reflections were broadened due to the size effect. The average grain size was 11.8nm according to the Scherrer formula, in agreement with HRTEM observation in Figure 10b.

The formation mechanism of GaN nanocomposites is not clear. A possible mechanism was proposed as follows [72]: (1) NH${}_{4}$Cl is very soluble in liquid ammonia (124g/100g NH${}_{3}$) and the resulting solution possesses a strong acidity; (2) Metal gallium reacts with ammonia to produce Ga${}^{3+}$ ions and form soluble intermediate compounds such as Ga(NH${}_{3}$)${}_{n}$Cl${}_{3}$ (n=1-14) [127] in such a acidic solution; (3) Then nano-sized GaN particles would precipitate from either the decomposition or further reactions of these intermediate compounds with liquid ammonia; (4) There was a strong aggregating tendency for nanosized clusters in the solution and the GaN nanocrystals were consolidated into GaN nanocomposites when the pressure in an autoclave is high enough.

## 5. Optical Properties of GaN Self-assemblies

The GaN self-assemblies talked here are consisted of nanoparticles whose optical properties strongly depend on crystalline size. Their photoluminescence and Raman scattering are discussed in this section.

### 5.1. Photoluminescence

**Figure 11:**
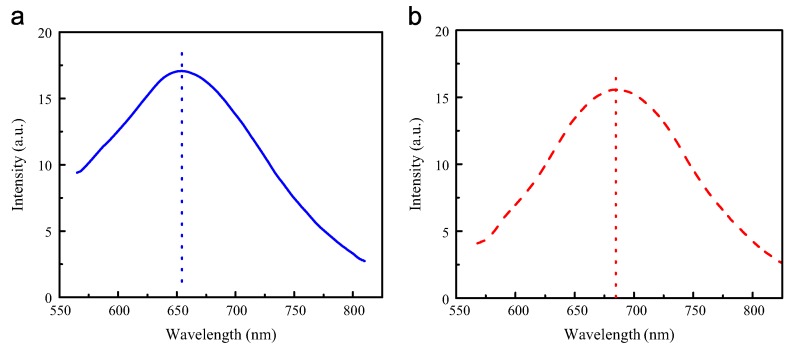
Photoluminescence spectra of (**a**) a GaN circular microtube with wurtzite structure and (**b**) a GaN squared microtube with zinc-blende structure. Reprinted with permission from [123]. Copyright 2015 Elsevier.

Figure 11a shows a typical photoluminescence (PL) spectrum of an individual GaN circular microtube (Figure 7), excited by 532nm wavelength radiation. The laser beam was focused on the individual microtube under an optical microscope to excite the PL spectrum. A yellow band, centered at 653nm, was observed. Similar yellow bands were also observed in nanoparticles [23] and nanowires [32] with wurtzite structure.

Figure 11b shows a PL spectrum of the square microtubes shown in Figure 8. A strong and wide yellow band was observed at 550–750 nm. It is generally accepted that nitrogen vacancies and deep level impurities contribute to the yellow band.

**Figure 12:**
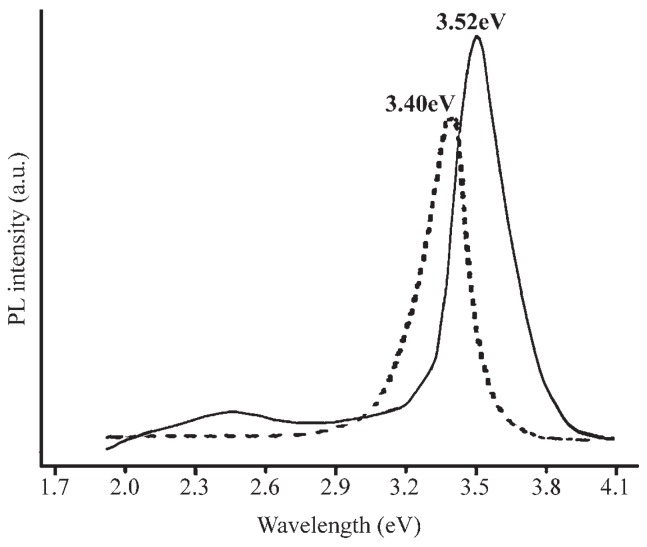
The room-temperature photoluminescence spectrum of hollow GaN spheres. Dashed line: bulk GaN. Reprinted with permission from [122]. Copyright 2005 Wiley.

Figure 12 shows a typical room-temperature PL spectrum of GaN nanospheres shown in Figure 4. The PL spectrum of GaN hollow spheres displayed a sharp ultraviolet near-band-edge transition at 3.52eV (352 nm). The observed strong band-edge emission blue-shifted ∼120meV relative to that of bulk GaN (dashed curve in the figure, 3.40eV). The blue-shift should be attributed to a quantum confinement effect. A weak yellow luminescence transition was observed at 2.3eV (539 nm) in the PL spectrum. The YL emission at 2.3 eV was located far below the band edge and was usually contributed to point defects within the GaN nanoparticles.

The PL spectrum of GaN nanotubes shown in Figure 6 was almost the same as that of GaN nanospheres.

Yellow bands were also observed in PL spectra of GaN nanocomposites [72]. The YL emission should come from the surface defects and vacancies of consisting GaN nanoparticles.

### 5.2. Raman Scattering

The space group of wurtzite GaN is $C_{6v}^{4}$ and two formula units are contained in its unit cell. According to the factor group analysis, there are six Raman-active modes, 1$A_{1}$(TO) + 1$A_{1}$(LO) + 1$E_{1}$(TO) + 1$E_{1}$(LO) + 2$E_{2}$.

**Figure 13:**
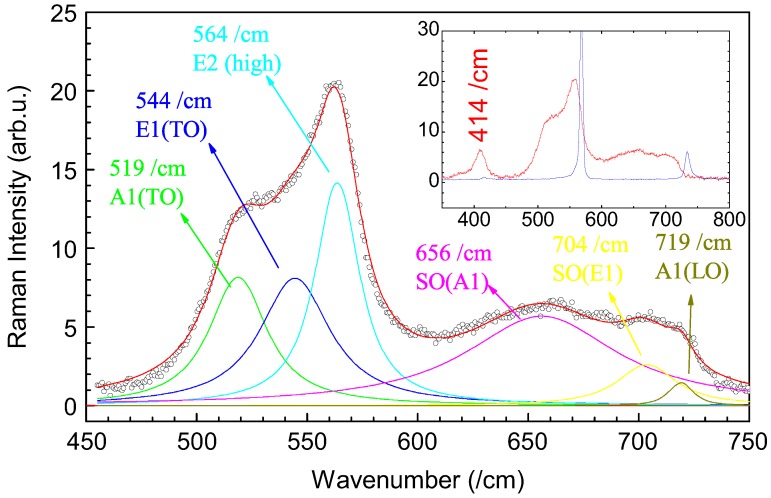
Room-temperature Raman scattering of a GaN circular microtubule. ∘: experimental; –: fitting. Inset: whole range of Raman scattering of the microtube (red curve) compared with that of GaN crystalline films (blue curve). Reprinted with permission from [123]. Copyright 2015 Elsevier.

Figure 13 shows a typical Raman spectrum of a GaN circular microtubes shown in Figure 7. The laser beam was focused on one GaN microtube under an optical microscope and the Raman spectrum was collected from the individual microtube. Among theoretically predicated six active Raman modes, four active modes were observed from the self-assemblies. The peaks at 519, 544, 564, and $719\;cm^{-1}$ corresponded to the A${}_{1}$(TO), E${}_{1}$(TO), E${}_{2}$(high), and A${}_{1}$(LO) symmetries, respectively. Compared with bulk data ($532\;cm^{-1}$ for A${}_{1}$(TO), $559\;cm^{-1}$ for E${}_{1}$(TO), $568\;cm^{-1}$ for E${}_{2}$(high), $734\;cm^{-1}$ for A${}_{1}$(LO)), the four active Raman modes significantly red-shifted to shorter wavenumbers. Two other Raman modes were observed on the low wavenumber side of the A${}_{1}$(LO) mode. The two peaks at $656\;cm^{-1}$ and $704\;cm^{-1}$ should be surface optical (SO) phonon modes corresponding to A${}_{1}$ and E${}_{1}$ symmetries respectively. The SO phonon modes should be caused by surface effects of the GaN nanorods. A unusual Raman peak was observed at $414\;cm^{-1}$ (inset of Figure 13). The peak should be the acoustic overtone from wurtzite GaN nanoparticles.

Table 2 compares the Raman modes of self-assembled GaN circular microtubes and GaN crystalline bulks. All the Raman-active modes red-shifted compared with crystalline bulks. A${}_{1}$(TO), E${}_{1}$(TO), and A${}_{1}$(LO) modes of the self-assemblies shifted more than these of individual GaN nanoparticles.

Raman modes of other kinds of free-standing self-assemblies also shifted as the circular microtubes.

## 6. Potential Applications of GaN Self-Assemblies

GaN self-assemblies of 1D nanomaterials (nanowires and nanorods) have been utilized as photocatalytic water splitting [129,130], photovoltaic devices [131,132], piezoelectric nanogenerators [65], and light-emitting diodes [63]. Free-standing GaN self-assemblies of 0D nanoparticles have larger surface/volume ratios than these of the GaN 1D nanomaterials. So the reviewed free-standing nanoporous self-assemblies should have wider potential applications.

### 6.1. Photocatalytic Water Splitting

GaN semiconductors have been employed for photoelectrochemical water splitting [133,134,135,136] since 2005 [133,134] to generate hydrogen gas. Up to now, GaN nanowire arrays have been utilized to generate hydrogen [129,130,137,138]. The highest incident-photon-to-current-conversion efficiency of 15%–18% [138] was obtained . Nanoporous GaN films were also employed to split water [139]. The nanoporous GaN films showed better efficiency and stability [139]. It was concluded that the large surface area of the porous GaN enhanced hole transport from photoanode to electrolyte and resulted better photovoltaic performances.

It was believed [138] that GaN nanostructures possess large surface-to-volume ratios, surface defects, rapid charge carrier separation, and enhanced optical absorption, being a better candidate to split water. Free-standing GaN porous assemblies shown in Figure 8, Figure 9 and Figure 10 have higher surface/volume ratio than all the reported nanowire/nanorod/nanoparticle arrays. Therefor a better photocatalytic performance is expected for free-standing GaN self-assemblies.

### 6.2. Piezoelectric Nanogenerators

GaN nanowire arrays produced output voltage pulses when pressed [65]. Theoretical calculation predicated [140] that piezoelectric constant of GaN nanomaterials increased with decreasing crystalline size of nanomaterials while surface piezoelectric coefficient was constant.

Crystalline size of free-standing GaN self-assemblies is several nanometers in three-dimensions, smaller than GaN nanowires that are in nanoscale in two dimensions. Therefore, the free-standing GaN self-assemblies of nanoparticles should have better piezoelectric performances than GaN nanowire arrays.

### 6.3. Thermoelectric Devices

GaN possesses excellent electronic transport properties such as high charge carrier mobility to be thermoelectric materials. In a GaN nanocrystalline ceramic prepared by hot pressing, a reasonably large Seebeck coefficient, *S*, of -58μV/K was reported at room temperature [141]. As self-assembly is a promising method for making nanostructured materials in situ, with proper optimization, the self-assembly materials hold promise for high efficiency downscaled thermoelectric devices [142].

Potential thermoelectric applications of the GaN-based low dimensional nanomaterials that are formed by self-assembly techniques are of great interest because of the possibility to be shaped into devices and circuits, and because of the potential for direct integration of microcooler/power generators with various electronic devices. Although to date there have not been reports on thermoelectric applications of self-assembled GaN and GaN-based nanomaterials, it is hoped that information on the improved thermoelectric properties of doped GaN-based bulk and low-dimensional nanomaterials present here may stimulate such development.

### 6.4. Other Potential Applications in Renewable Energy

GaN self-assemblies reviewed here are nanoporous and consisted of nanoparticles, holding novel optical properties. This kinds of nanomaterials should also have applications in batteries, capacitors, and solar cells. However, it is difficulty to massively produce such kinds of porous nanostructures. Few researches on renewable energy have been reported. More potential applications of the free-standing nanoporous nanomaterials are expected in the coming years.

## 7. Conclusions

The syntheses and physical properties of hexagonal GaN nanoparticles are briefly reviewed. These novel GaN nanoparticles can be self-assembled into free-standing nanospheres, nanotubes, microtubes, and composite bulks without any templates. The mechanism and physical properties of these self-assemblies are discussed. The potential applications of the assemblies are expected based on their high surface/volume ratios and tunable band-gaps.

## Figures and Tables

**Table 1:** Typical syntheses of gallium nitride (GaN) nanoparticles.

Method	Precursors	Catalysts	Reaction Temperature (${}^{\circ }C$)	Average Diameter (nm)	Reference
CVD	Ga	-	900–1150	5–8	[67]
nitridation	Ga${}_{2}$O${}_{3}$	-	800–1100	40–500	[68]
	GaSb	-	900	14–23	[69]
	Ga(NO${}_{3}$)${}_{3}$	-	850	5	[70]
	GaO(OH)	-	900–1000	12–15	[71]
solvothermal	Ga ${}^{†}$	Li	350–500	30–70	[23]
	Ga ${}^{†}$	NH${}_{4}$I	350–500	12	[72]
	GaCl${}_{3}$${}^{‡}$	Li${}_{3}$N	280	30	[73]
ball milling	Ga and NH${}_{3}$	-	100	10–30	[74]

^†^: ammono-thermal; ^‡^: benzene-thermal.

**Table 2:** Raman scattering of hexagonal GaN materials.

Mode	E${}_{2}$ (low)	A${}_{1}$(TO)	E${}_{1}$(TO)	E${}_{2}$(high)	SO(A1)	SO(E1)	A${}_{1}$(LO)	E${}_{1}$(LO)	Reference
	(cm${}^{-1}$)	(cm${}^{-1}$)	(cm${}^{-1}$)	(cm${}^{-1}$)	(cm${}^{-1}$)	(cm${}^{-1}$)	(cm${}^{-1}$)	(cm${}^{-1}$)	
crystals	144	533	561	569	-	-	735	743	[128]
nanoparticles †	-	528	552	564	656	-	730	-	[88]
microtubes ‡	-	519	544	564	656	704	719	-	[123]

SO: surface optical phonon mode. †: crystalline size: ∼12 nm. ‡: crystalline size: ∼100 nm.
